# Supplementary material for: Flutter to tumble transition of buoyant spheres triggered by rotational inertia changes
Source: Nat Commun. 2018 May 4;9:1792. doi: 10.1038/s41467-018-04177-w (PMC5935758; doi:10.1038/s41467-018-04177-w)
Supplement: Supplementary file 1 — Supplementary Information [file 41467_2018_4177_MOESM1_ESM.pdf]

SUPPLEMENTAL INFORMATION

FLUTTER TO TUMBLE TRANSITION OF BUOYANT SPHERES TRIGGERED BY ROTATIONAL INERTIA CHANGES

by Mathai et al.

SUPPLEMENTARY FIGURES

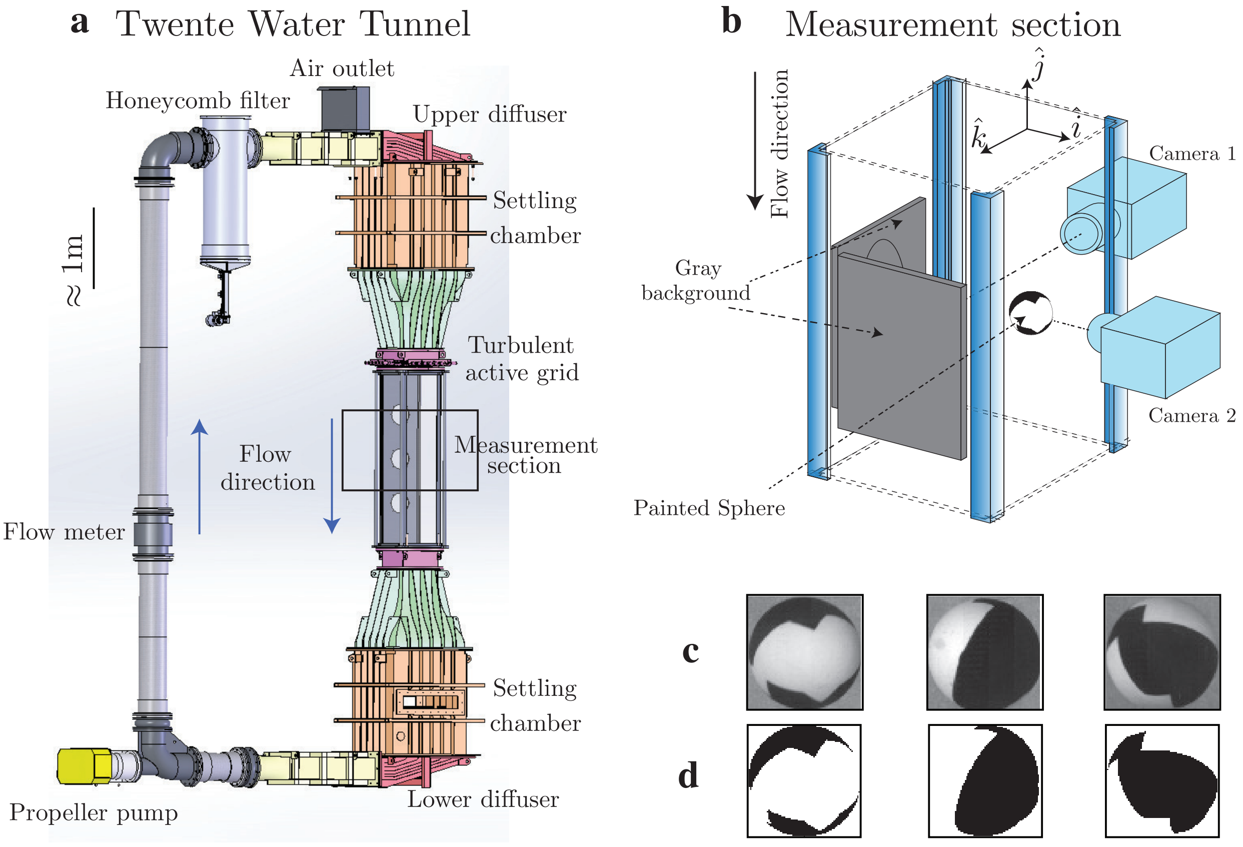

Supplementary Figure 1. Experimental facility and sphere rotation tracking method used in the present study. (a) Drawing of the Twente Water tunnel (TWT) facility, used to generate a nearly homogeneous and isotropic turbulent flow in the measurement section (shown by the rectangular selection). Spherical particles were released within this turbulent flow. (b) Experimental arrangement involving two orthogonally placed cameras and a painted spherical particle. The flow direction is downward, and the buoyant particle either gently rises or settles with respect to the laboratory coordinate system $\hat{i}\hat{j}\hat{k}$. (c) Experimental images of the painted sphere for arbitrary orientations, and (d) the synthetic image equivalents, as found by the orientation detection program.

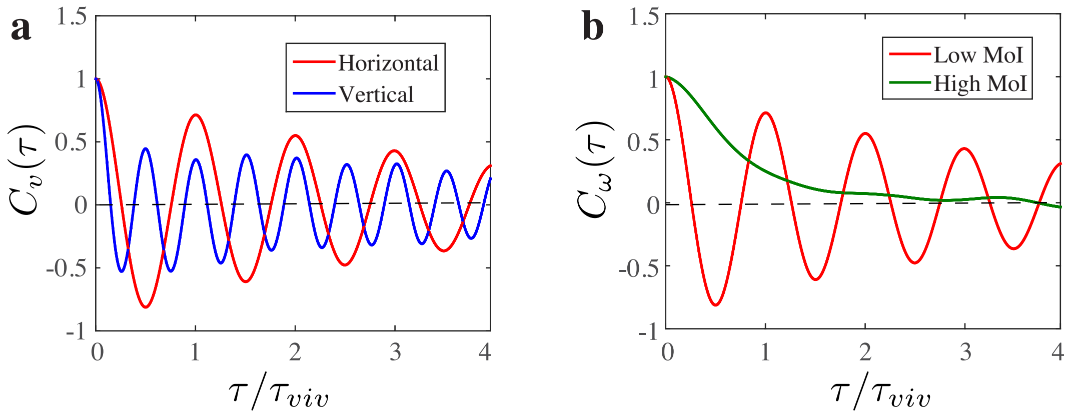

Supplementary Figure 2. Autocorrelation functions of velocity and angular velocity components. (a) Autocorrelation of the horizontal (red) and vertical (blue) velocity components of the low MoI sphere in turbulent flow. The horizontal axis is normalized by the vortex-shedding based time-scale $\tau_{viv} \propto$ D/U, where D is the diameter of the sphere and U is the mean rise velocity of the particle. The vertical velocity fluctuations have twice the frequency of the horizontal velocity fluctuations, a well-known characteristic of vortex-induced vibrations[1]. (b) Lagrangian temporal autocorrelation of a horizontal component of angular velocity for the high (green colour) and low (red colour) MoI spheres in turbulent flow. For the low MoI sphere, the angular velocity shows oscillations at the same frequency as the linear velocity, shown in (a), i.e. the fluttering motion occurs in synchronization with the particle's rotational motion. For the high MoI sphere, the angular velocity decorrelates gradually and without periodicity.

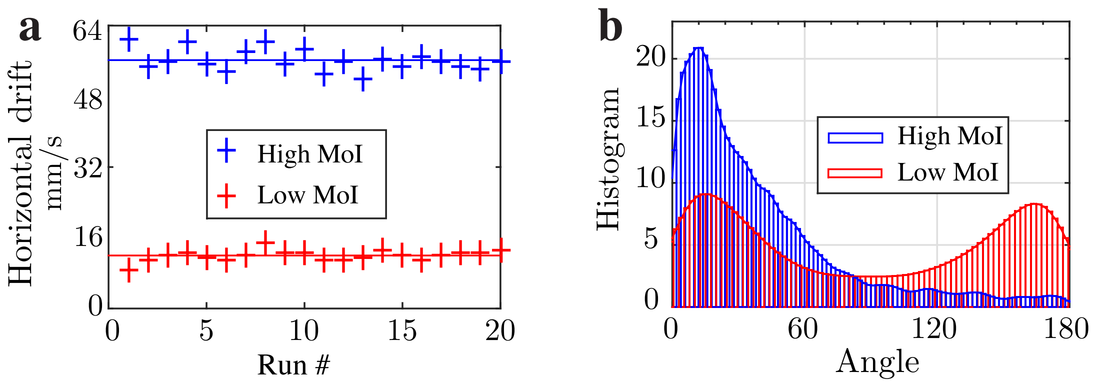

Supplementary Figure 3. Mean horizontal drift and its alignement with the intantaneous particle rotation for buoyant spheres in a turbulent flow. (a) Mean horizontal drift velocities of the high and low MoI spheres. (b) Histogram of the angle between the horizontal drifting direction and the vector $\vec{\omega} \times \hat{j}$, where $\hat{j}$ is the unit vector in the vertical direction. The high MoI sphere shows alignment. The low MoI sphere shows two peaks at around 0° and 180°, indicating alignment and anti-alignment. Movies showing the motion of the two spheres are given in Supplementary movies 1 & 2.

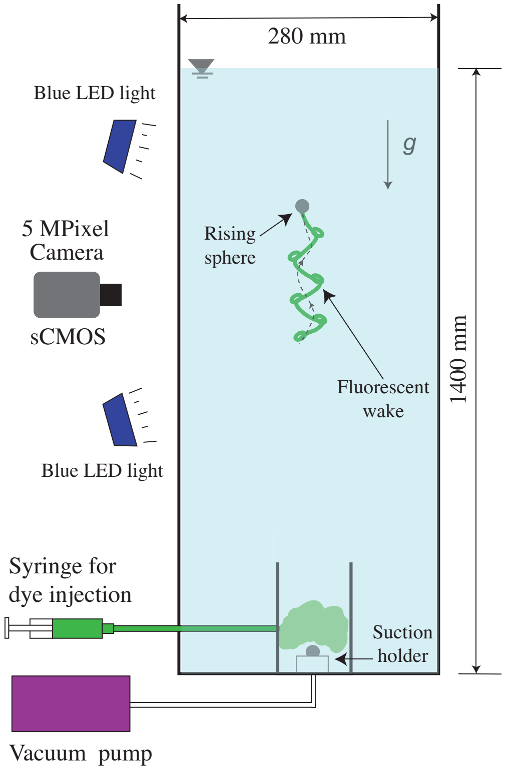

Supplementary Figure 4. Experimental arrangement used for visualizing the trajectories and wakes of freely rising spheres. Fluorescein sodium dye at 5×10^{-3}mol/l concentration is injected at the bottom of the water tank. The sphere is held at the bottom by applying suction.

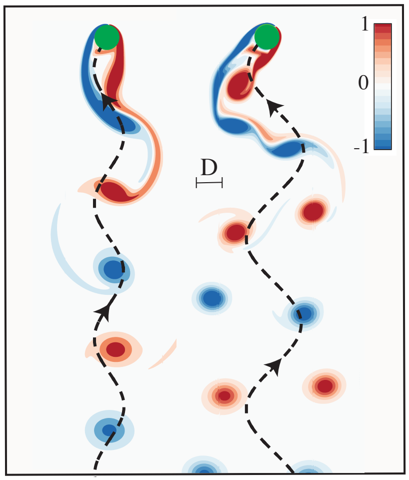

Ga = 500

Supplementary Figure 5. Trajectories and wakes of freely rising cylinders with mass density ratio $\Gamma = 0.1$. The left case shows the trajectory and wake of a rising cylinder with a contraint on rotation. It rises with a small amplitude of transverse oscillation and sheds 2-Single (2S) vortices per cylinder of oscillation. The right case shows the same cylinder with the rotation constraint removed ($I^* = 0.1$). In this case we observe large amplitude oscillations. The wake shows 2-Pairs (2P) of vortices shed per cycle of oscillation. These patterns may be considered analogous to the 2-Ring and 4-Ring vortex modes observed for freely rising spheres.

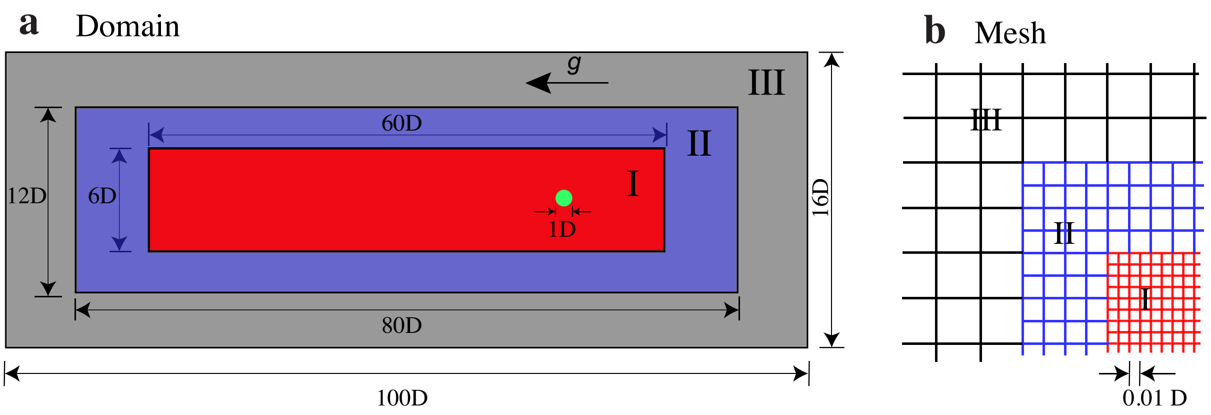

Supplementary Figure 6. Domain of the mesh used in the numerical simulations. (a) Full domain, where D denotes the diameter of the circular cylinder. From region I to II to III, the mesh coarsens. (b) Mesh coarsening from region I to II to III. g denotes the direction of gravity, and the particle stays within region I in the simulations.

SUPPLEMENTARY DISCUSSION

The Kelvin-Kirchhoff equations for a rigid particle in a flow

A minimalistic description of the motion of a rigid body in a flow requires consideration of the unsteady fluid forces and torques acting on the body[2,3]. The classical Kelvin-Kirchhoff equations expressing linear and angular momentum conservation for a spherical particle in an incompressible flow reduces to:

$$(\Gamma + \frac{1}{2} + \mathrm{B_U}\ \delta)\ \frac{\mathrm{d}\mathbf{U}}{\mathrm{dt}} + \Gamma\ \mathbf{\Omega} \times \mathbf{U} = \frac{\mathbf{F}_Q}{m_f} + (\Gamma - 1)g; \quad (1)$$

$$(\frac{1}{10}\mathrm{I}^* + \mathrm{B}_\Omega\ \delta)\frac{\mathrm{d}\mathbf{\Omega}}{\mathrm{dt}} = \frac{\mathbf{T}_Q}{m_f D^2}; \quad (2)$$

where Γ is the sphere mass density ratio, $\mathbf{U}$ is the sphere velocity vector, $\mathbf{\Omega}$ is the angular velocity vector, g is the acceleration due to gravity, $\mathrm{I}^* \equiv \mathrm{I}_p/\mathrm{I}_f$ is the moment of inertia ratio, where I_p is the particle moment of inertia, and I_f is the moment of inertia of the fluid volume displaced by the particle, $\mathbf{F}_Q$ and $\mathbf{T}_Q$ are the fluid force and torque vectors, respectively, m_f is the mass of the fluid displaced by the sphere, and D is the sphere diameter. Note that $\delta = \sqrt{\frac{\nu\tau}{\pi D^2}}$ is the dimensionless Stokes boundary layer which develops in a time τ[4,5]. The prefactors $\mathrm{B_U} = 18$ and $\mathrm{B}_\Omega = 2$ are known analytically from the unsteady viscous contributions[3,6].

Equations 1 & 2 point to two parameter dependences, namely, the particles mass and its moment of inertia. In addition, the rotation rate of the particle $\mathbf{\Omega}$ is linked to I^*, and couples with eq. 1 through a force term. This coupling could be important only if the torques acting on the particle are significant. This is indeed the case for anisotropic particles (disks, strip, and rods), where significant rotational motions have been reported in prior studies[7,8]. For anisotropic particles, the body orientation with respect to the flow determines both the fluid forces and torques. Consequently, a major part of the torque comes from the pressure forces on the particle. In contrast, for isotropic particles (spheres and circular cylinders), only skin friction forces $\mathbf{F_s}$ could contribute to the torque on the body, i.e. $\mathbf{T}_Q = \oint \mathbf{r} \times \mathbf{F_s}\ dA$. For a sphere at moderate Reynolds numbers, the skin friction forces are small as compared to the pressure forces. Therefore, the role of rotation has generally been ignored for spherical particles.

The torque balance equation given by eq. 2 may be used to obtain an estimate of the typical rotational motion for a sphere in a flow. This would first require an estimate of the Stokes boundary layer thickness δ, which grows during a time scale τ. The largest time scales for a sphere in a flow are set by the vortex shedding behind the sphere. Therefore, $\tau \sim t_v$, where t_v is the half period of shedding of a sphere. At moderate Reynolds numbers ($\mathrm{Re}_p \sim \mathcal{O}(10^3)$), the typical half period of vortex shedding[9]: $t_v \approx \frac{2U}{D}$. This implies that $\delta \approx \frac{k_1}{\sqrt{\mathrm{Re}_p}}$, where $k_1 = 20\sqrt{2/\pi}$, and $\mathrm{Re}_p = UD/\nu$ is the mean particle Reynolds number. Non-dimensionalizing eq. 2 in terms of U and D, we obtain:

$$(\mathrm{I}^* + k_1 \frac{1}{\sqrt{\mathrm{Re}_p}}) \frac{\Delta\zeta}{\Delta\tilde{t}^2} = k_2 \mathrm{C}_\tau. \tag{3}$$

Here, I^* is the moment of inertia ratio , $C_\tau = \frac{4|\mathrm{T}_Q|}{3U^2}$ is the torque coefficient[10], $\Delta\zeta$ is the rotational amplitude, and $k_2 = 7.5$ is a prefactor[6,11].

The largest rotational amplitude $\Delta\zeta$ is expected when $\mathrm{I}^* \to 0$. For $\mathrm{Re}_p \sim \mathcal{O}(10^3)$, and $\mathrm{C}_\tau \sim 10^{-4}$, eq. 3 yields an estimate for the rotational amplitude $\Delta\zeta \approx 0.34^\circ$. Such small rotations were expected to not influence the instability onset[12]. This has prompted many investigators to vary the particle's mass density ratio by using hollow and solid spheres, made from a variety of materials[13–16]. In doing so, the particle's moment of inertia (MoI or I) varied erratically, the implications of which have not been considered.

Experiments

The turbulent flow experiments were performed in the Twente Water Tunnel facility, in which an active grid generated nearly homogeneous isotropic turbulence in the measurement section. In Supplementary Fig. 1(a) & (b), we show a schematic of the Twente Water Tunnel (TWT) facility along with the experimental arrangement used for tracking the translation and rotation of the sphere simulateneously. The Taylor Reynolds number of the flow $\mathrm{Re}_\lambda \approx 300$, and the ratio of particle size to dissipative length scale $\Xi \approx 100$. A downward flow in the measurement section, at a mean flow speed comparable to the mean rise-velocity of the spheres enabled us to track the three-dimensional trajectories of the rising spheres for a significant duration.

Sphere orientation detection

The spherical particles were first painted with an analytically prescribed pattern and released in the flow. The pattern was composed of simply connected black and white areas. Two cameras were placed at a 90 degree angle between them, and front-lighting was provided from eight 20W LED lights sources. The LEDs were positioned in such a way that the pattern was well-lit throughout the measurement window. A gray background was used to ensure sufficient contrast with both the black and the white parts of the pattern. In the image processing step, we inverted the dark regions of the images corresponding to the painted pattern. Thus, a full circle was visible for accurate center detection. Following this step, the spheres were detected using the Circular Hough Transform method. Once the center and radius were determined, the image of the sphere was cropped for the pattern-detection step (Supplementary Fig. 1(c)). The image was binarized for comparison with the synthetic images. The synthetic image was constructed based on the analytic description of the pattern and an orientation input. Given a coordinate on the surface of the sphere, the function returns either a '0' or a '1' depending on the color of its corresponding infinitesimally small surface element. The sum of the absolute difference between the binarized image pixels and the corresponding pixels of the synthetic image was used as a measure of the mismatch between the image and a synthetic orientation candidate. The orientation for which this comparison yields a minimum is determined using a Nelder-Mead minimization algorithm[17]. Supplementary Fig. 1(d) shows one to one comparisons between the synthetic image outputs obtained by the minimization algorithm corresponding to the experimental images provided by the user in Supplementary Fig. 1(c).

Tumbling vs fluttering dynamics

Supplementary Fig. 2(a) shows the Lagrangian autocorrelation function of velocity $C_v(\tau)$ for the low moment of inertia (MoI) sphere in turbulent flow. The function shows strong oscillations for both the horizontal (red) and the vertical (blue) components of velocity. Importantly, the frequency of horizontal fluctuations is half that of the vertical fluctuations and matches roughly the vortex shedding frequency $f_{viv} = 1/\tau_{viv}$. The same behavior is observed for the high MoI sphere, although, with a marginal decrease in f_{viv}. Next we com-

pare the Lagrangian autocorrelation of one of the horizontal components of angular velocity $C_\omega(\tau)$ (Supplementary Fig. 2(b)). For the low MoI sphere (red), we see oscillations at a frequency which exactly matches the frequency of the translational velocity given in Supplementary Fig. 2(a). Thus, the rotational motion appears to be in synchronization with the translation of the sphere. However, for the high MoI sphere, the rotational autocorrelation $C_\omega(\tau)$ is dramatically different. $C_\omega(\tau)$ gradually decreases to zero without showing any oscillations. The time scale of this decorrelation is significantly larger than the vortex shedding timescale. Thus the translation and rotation do not appear to be synchronized for the high MoI sphere.

The mean horizontal drifting rates for the two spheres are shown in Supplementary Fig. 3(a). Each datapoint corresponds to a separate experimental run, composed of a long and continious trajectory of the particle. The high MoI sphere shows a significant horizontal drift rate of about 55 mm/s, which is nearly five times that of the low MoI sphere (≈ 11 mm/s). From Supplementary movie 1, we observe that the high MoI sphere tumbles in the flow with respect to this mean drifting direction. Statistical evidence for this may be seen when we compare the horizontal drifting direction with the direction of rotation. In Supplementary Fig. 3(b), we plot the angle between $\vec{\omega} \times \hat{j}$ and the mean horizontal drift direction. Here, $\vec{\omega}$ is the angular velocity vector, and $\hat{j}$ is the unit vector in the vertical direction (Supplementary Fig. 1(b)). $0°$ indicates alignment, and $180°$ indicates anti-alignment. The high MoI sphere shows alignment between the two vectors, indicating that the sphere (statistically) tumbles in the flow. Upon reducing the moment of inertia, the sphere flutters in the flow, as signified by the two peaks around $0°$ and $180°$(evident also from Supplementary movie 2). We believe that the tumbling motion of the high MoI sphere aids it to drift horizontally in the flow, while the fluttering motion of the low MoI sphere stabilizes it to remain in the middle of the measurement section. A direct analogy may be drawn to the motions reported for falling disks, strips and paper (falling 2D strips in Supplementary movie 3), where a moment-of-inertia reduction triggers a tumble-flutter transition[8]. A geometrical anisotropy of the rising/falling particle was considered necessary to induce this transition. The present observations for spheres suggests that this geometrical anisotropy factor may not be necessary for inducing this transition.

Dye visualization experiment

Supplementary Fig. 4 shows the experimental setup used for visualizing the trajectories and wakes of the freely rising spheres. The setup consists of a glass tank with dimensions 280 mm $\times$ 280 mm wide, and 1500 mm high. The tank was filled with either water or a mixture of glycerine and water. The viscosity of the mixture was monitored at regular intervals using a rheometer. The sphere was held to the bottom of the tank by applying suction, and fluorescent dye (fluorescein-sodium) was injected into the cylindrical container just above the sphere. The dye was prepared with a concentration $\approx 5 \times 10^{-3}$ mol/L of fluorescein-sodium. The dye has a molecular diffusion coefficient in water, $D_m = 4 \times 10^{-10}$ m^2/s at 22 °, which is much smaller than the water kinematic viscosity ν. This leads to a high Schmidt number, $Sc \equiv \nu/D_m \approx 2500$[18]. The tank was left undisturbed for one hour. The glass tank was illuminated by eight blue LED floodlights, with a wavelength $\approx$ 470 nm. This corresponds to a peak in the emission spectrum of the fluorescent dye. A camera was positioned orthogonally facing a side-wall of the glass tank. A light filter placed in front of the camera ensured that the incident blue light from the LED lamps was filtered out. The sphere was gently released by withdrawing the suction. The sphere rose through the liquid and entrained the dye on its way up. The entrained dye in the wake was slowly shed as the sphere rose, enabling us to visualize the wake. This gave a qualitative picture of the wake structure. The experiment was performed at two Galileo numbers: Ga $\approx$ 6000 and Ga $\approx$ 500. At high Ga, the wakes diffuse quickly, while at lower Ga, the vortex rings shed by the wakes are clearly visible. Supplementary movies 4 & 5 show the wake patterns at these Ga.

Numerical simulations of freely rising cylinders

An interesting analogy may be drawn between a rising spherical particle, and its 2D equivalent: a rising circular cylinder. For instance, a rising sphere at moderate Ga ($\approx$ 1000) sheds four rings and two vortex rings per cycle of oscillation (4-R and 2-R vortex modes)[19]. Analogous to this, a rising cylinder sheds four vortices and two vortices per cycle of oscillation[20]. Motivated by this analogy, we had recently performed direct numerical simulations (DNS) of the two-dimensional motion of rising circular cylinders in quiescent

fluid[21]. The fluid motion is governed by the incompressible Navier-Stokes equations:

$$\frac{\partial \mathbf{u}}{\partial t} + \mathbf{u} \cdot \nabla \mathbf{u} = \nabla p + \frac{\mathbf{1}}{Re} \nabla^{\mathbf{2}} \mathbf{u} + \mathbf{f}, \tag{4}$$

where $\mathbf{u}$ is the velocity vector, p is the pressure, Re is the Reynolds number, and $\mathbf{f}$ is the Eulerian body-force that is used to mimic the effects of the immersed body on the flow[22,23]. The solver uses a discrete stream-function formulation for the incompressible Navier-Stokes equations[24], and a virtual-force implementation[25] enables us to deal with both light and heavy particles. The code has been extensively validated[22,23,26]. Supplementary Fig. 5 compares the trajectories (and wakes) of two buoyant cylinders, with and without a constraint on rotation. Upon removing the rotation constraint, the amplitude of trajectory in increased, along with a change in the wake pattern. The rectangular computational domain used in these simulations has a size 100D $\times$ 16D (Supplementary Fig. 6(a)). The mesh width was 0.01D in region I, where D is the cylinder diameter. The cylinder motion was localized to this domain. Gradual coarsening of mesh was adopted in regions II and III (Supplementary Fig. 6(b)).

SUPPLEMENTARY REFERENCES

[1] C. Williamson and R. Govardhan, Vortex-induced vibrations, Annu. Rev. Fluid Mech. **36**, 413 (2004).

[2] P. Ern, F. Risso, D. Fabre, and J. Magnaudet, Wake-induced oscillatory paths of bodies freely rising or falling in fluids, Annu. Rev. Fluid Mech. **44**, 97 (2012).

[3] F. Auguste and J. Magnaudet, Path oscillations and enhanced drag of light rising spheres, J. Fluid Mech. **841**, 228 (2018).

[4] H. Schlichting, K. Gersten, E. Krause, and H. Oertel, Boundary-layer theory (Springer, ADDRESS, 1955), Vol. 7.

[5] P. Kundu and I. Cohen, Fluid mechanics. 2004, Elsevier Academic Press, San Diego). Two-and three-dimensional self-sustained flow oscillations **307**, 471 (2008).

[6] W. Zhang and H. Stone, Oscillatory motions of circular disks and nearly spherical particles in viscous flows, J. Fluid Mech. **367**, 329 (1998).

[7] S. B. Field, M. Klaus, M. G. Moore, and F. Nori, Chaotic dynamics of falling disks, Nature **388**, 252 (1997).

[8] A. Belmonte, A. Eisenberg, and E. Moses, From flutter to tumble: inertial drag and Froude similarity in falling paper, Phys. Rev. Lett. **81**, 345 (1998).

[9] E. Achenbach, Vortex shedding from spheres, J. Fluid Mech. **62**, 209 (1974).

[10] G. Bouchet, M. Mebarek, and J. Dušek, Hydrodynamic forces acting on a rigid fixed sphere in early transitional regimes, European Journal of Mechanics-B/Fluids **25**, 321 (2006).

[11] R. Gatignol, The Faxén formulas for a rigid particle in an unsteady non-uniform stokes-flow, J. Mec. Theor. Appl. **2**, 143 (1983).

[12] M. Jenny, J. Dušek, and G. Bouchet, Instabilities and transition of a sphere falling or ascending freely in a Newtonian fluid, J. Fluid Mech. **508**, 201 (2004).

[13] M. Horowitz and C. H. K. Williamson, The effect of Reynolds number on the dynamics and wakes of freely rising and falling spheres, J. Fluid Mech. **651**, 251 (2010).

[14] A. W. Preukschat, Ph.D. thesis, California Institute of Technology, 1962.

[15] C. H. J. Veldhuis, A. Biesheuvel, and D. Lohse, Freely rising light solid spheres, Int. J. Mult. Flow **35**, 312 (2009).

[16] D. G. Karamanev, C. Chavarie, and R. C. Mayer, Dynamics of the free rise of a light solid sphere in liquid, AIChE journal **42**, 1789 (1996).

[17] V. Mathai, M. W. M. Neut, E. P. van der Poel, and C. Sun, Translational and rotational dynamics of a large buoyant sphere in turbulence, Exp. Fluids **57**, 1 (2016).

[18] E. Alméras, F. Risso, V. Roig, S. Cazin, C. Plais, and F. Augier, Mixing by bubble-induced turbulence, J. Fluid Mech. **776**, 458 (2015).

[19] M. Horowitz and C. H. K. Williamson, Critical mass and a new periodic four-ring vortex wake mode for freely rising and falling spheres, Phys. Fluids **20**, 101701 (2008).

[20] M. Horowitz and C. H. K. Williamson, Vortex-induced vibration of a rising and falling cylinder, J. Fluid Mech. **662**, 352 (2010).

[21] V. Mathai, X. Zhu, C. Sun, and D. Lohse, Mass and moment of inertia govern the transition in the dynamics and wakes of freely rising and falling cylinders, Phys. Rev. Lett. **119**, 054501 (2017).

[22] X. Zhu, G. He, and X. Zhang, Flow-mediated interactions between two self-propelled flapping filaments in tandem configuration, Phys. Rev. Lett. **113**, 238105 (2014).

[23] X. Zhu, G. He, and X. Zhang, An improved direct-forcing immersed boundary method for fluid-structure interaction simulations, J. Fluids Eng. **136**, 040903 (2014).

[24] X. Zhu, G. He, and X. Zhang, Numerical study on hydrodynamic effect of flexibility in a self-propelled plunging foil, Comp. Fluids **97**, 1 (2014).

[25] S. Schwarz, T. Kempe, and J. Fröhlich, A temporal discretization scheme to compute the motion of light particles in viscous flows by an immersed boundary method, J. Comp. Phys. **281**, 591 (2015).

[26] X. Zhu, G. He, and X. Zhang, How flexibility affects the wake symmetry properties of a self-propelled plunging foil, J. Fluid Mech. **751**, 164 (2014).
