# Supplementary material for: Flutter to tumble transition of buoyant spheres triggered by rotational inertia changes
Source: Nat Commun. 2018 May 4;9:1792. doi: 10.1038/s41467-018-04177-w (PMC5935758; doi:10.1038/s41467-018-04177-w)
Supplement: Supplementary file 3 — Description of Additional Supplementary Files [file 41467_2018_4177_MOESM3_ESM.pdf]

Description of Additional Supplementary Files

File Name: Supplementary Movie 1
Description: Typical trajectories of the high moment of inertia (MoI) sphere in a turbulent flow. Note that a mean downward flow roughly matches the sphere rise velocity, enabling us to observe these motions for a long duration.

File Name: Supplementary Movie 2
Description: Typical trajectory of the low moment of inertia (MoI) sphere in a turbulent flow. Note that a mean downward flow roughly matches the sphere rise velocity, enabling us to observe these motions for a long duration.

File Name: Supplementary Movie 3
Description: Tumbling and fluttering motions for a high MoI strip (left) and a low MoI strip (right), respectively, falling in still fluid.

File Name: Supplementary Movie 4
Description: Free-rise trajectories and wakes of the high MoI (left) and low MoI (right) spheres rising in still water at $Ga \approx 6000$

File Name: Supplementary Movie 5
Description: Free-rise trajectories and wake of high MoI (left) and low MoI (right) spheres rising in 2.3:1 by volume mixture of glycerine and water at $Ga \approx 500$.

File Name: Supplementary Movie 6
Description: Two-dimensional trajectories and wakes of a buoyant circular cylinder with (left) and without (right) a constraint on rotation. Here $Ga = 500$.
